# The molecular landscape of breast mucoepidermoid carcinoma

**DOI:** 10.1002/cam4.5754

**Published:** 2023-03-14

**Authors:** Konstantinos Venetis, Elham Sajjadi, Mariia Ivanova, Silvia Andaloro, Simona Pessina, Chiara Zanetti, Alberto Ranghiero, Gabriele Citelli, Chiara Rossi, Marco Lucioni, Umberto Malapelle, Fabio Pagni, Massimo Barberis, Elena Guerini‐Rocco, Giuseppe Viale, Nicola Fusco

**Affiliations:** ^1^ Division of Pathology IEO, European Institute of Oncology IRCCS Milan Italy; ^2^ Department of Oncology and Hemato‐Oncology University of Milan Milan Italy; ^3^ Division of Anatomic Pathology, Department of Molecular Medicine, Fondazione IRCCS Policlinico San Matteo University of Pavia Pavia Italy; ^4^ Department of Public Health University Federico II Naples Italy; ^5^ Department of Medicine and Surgery Pathology, University Milan Bicocca Milan Italy

**Keywords:** biomarkers, breast cancer, diagnosis, molecular profiling, mucoepidermoid carcinoma, rare tumors, triple‐negative breast cancer

## Abstract

Mucoepidermoid carcinoma (MEC) of the breast is an extremely rare salivary gland‐type tumor characterized by epidermoid, basaloid, intermediate, and/or mucinous cells arranged in solid and cystic patterns. Despite their triple‐negative phenotype, breast MECs are generally considered low‐risk malignancies but their biology is largely unexplored; therefore, guidelines for clinical management are lacking. Here, we sought to characterize the molecular landscape of breast MECs. Thirteen cases were histologically reviewed, characterized for tumor‐infiltrating lymphocytes (TILs), and were subjected to immunohistochemistry for programmed death‐ligand 1 (PD‐L1, clone 22C3), EGFR, and amphiregulin (AREG). Rearrangements in *MAML2* and *EWSR1* were investigated by fluorescent in situ hybridization. Targeted next‐generation sequencing of 161 genes was performed on eight cases. Most MECs had low histological grade (*n* = 10, 77%), with the presence of TILs (*n* = 9/12; 75%) and PD‐L1 combined positive score ranging from 10 to 20 (*n* = 4/6; 67%). All cases showed EGFR and AREG overexpression and were fusion negative. Enrichment of genetic alterations was observed in PI3K/AKT/mTOR and cell cycle regulation pathways, while only one case harbored *TP53* mutations. This is the first study providing extensive molecular data on breast MECs and the largest collection of cases available to date in the literature. Breast MECs lack *TP53* mutations found in high‐grade forms of triple‐negative breast cancers and *MAML2* or *EWSR1* rearrangements pathognomonic of salivary MECs. Triple‐negativity and PD‐L1 positivity suggest a window of opportunity for immunotherapy in these patients. The EGFR/AREG axis activation, coupled with the mutational patterns in PI3K/AKT/mTOR and cell cycle pathways warrants caution in considering MECs as low‐risk neoplasms.

## Graphical abstract

Here, we provide the most comprehensive molecular landscape of the largest collection of breast mucoepidermoid carcinoma cases available in the literature to date, reporting the results of immunohistochemistry, next‐generation sequencing and fluorescent in‐situ hybridization. Our analyses confirm that not all these tumors should be regarded as low‐grade triple‐negative breast cancers and therefore warrant deep‐dive research to support individualized treatment strategies.

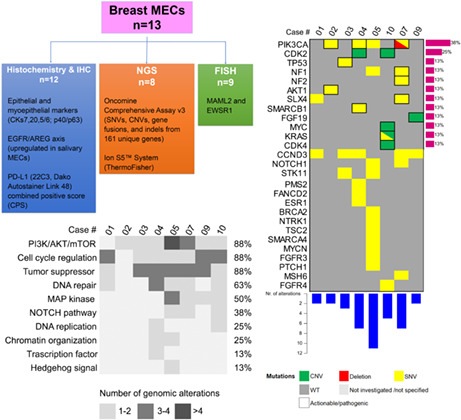

## 1 INTRODUCTION

Mucoepidermoid carcinoma (MEC) of the breast is an exceedingly rare type of salivary gland‐like tumor characterized by an admixture of basaloid, intermediate (i.e., clear cells), squamoid, and mucinous cells arranged in solid and cystic growth patterns within a variable myxoid stroma.^1^, ^2^ Due to the lack of estrogen receptor (ER), progesterone receptor (PgR), and HER2 expression, this tumor belongs to the spectrum of triple‐negative breast cancers (TNBCs).^3^ Similar to other salivary gland‐like breast malignancies, MECs are reported to have a less aggressive clinical behavior than the archetypal TNBC.^4^, ^5^ Regrettably, since the first description by Patchefsky et al. in 1979,^6^ only a handful of cases have been reported in the literature.^6^, ^7^, ^8^, ^9^

To identify diagnostic and actionable biomarkers, researchers have been looking for similarities between primary breast MECs and those arising in the salivary glands. In salivary MECs, the most frequent molecular alteration is the t(11, 19)(q14–21; p12–13) translocation, resulting in the oncogenic CREB‐regulated transcription coactivator 1 (*CRTC1*)‐mastermind‐like protein 2 (*MAML2*) fusion transcript (less commonly *CRTC3‐MAML2*).^10^ This structural rearrangement induces the overexpression of amphiregulin (AREG), an epidermal growth factor receptor (EGFR) ligand, via co‐activation of the transcription factor cAMP response element‐binding protein (CREB).^11^ As a consequence, the oncogene EGFR is often overexpressed in these tumors.^12^ Of note, both AREG and EGFR upregulation are involved in proliferation and metastasis in TNBC.^13^, ^14^ Patients with salivary gland and lung MECs harboring *CRTC1‐MAML2* fusion have a significantly lower risk of local recurrence, metastases, or tumor‐related death compared with those with tumors lacking the fusion protein.^15^ A subset of fusion‐negative salivary gland MECs were found to host (EWS RNA binding protein 1) *EWSR1* mutations, and it has been proposed to reclassify these tumors as hyalinizing clear cell carcinoma, which has a relatively better prognosis.^16^, ^17^ Among the reported cases of breast MEC that have been investigated for the presence of *MAML2* rearrangements, only a three of them presented this feature, questioning the diagnostic role of this biomarker in the breast.^18^, ^19^

Notwithstanding previous efforts to characterize breast MEC by morphology and ancillary studies, no pathognomonic molecular features have been identified. Owing to their rarity, the biology of breast MECs is largely unexplored, and they pose diagnostic challenges; therefore, their clinical management lacks widely adopted guidelines. In this study, we sought to characterize the histopathological characteristics and the repertoire of somatic genetic alterations and the tumor immune microenvironment characteristics of a large collection of breast MECs.

## 2 MATERIALS AND METHODS

### 2.1 Patients and tissue specimens

This study was approved by the local Ethics Committee under the approval number #UID3472; written informed consent was obtained from patients for use of tissue samples. All patients were diagnosed and managed at the European Institute of Oncology IRCCS (IEO), Milan and Fondazione IRCCS Policlinico San Matteo, Pavia, Italy between 1994 and 2021. Taken together, 13 breast MECs were included in this study and subsequently revised, re‐classified, and re‐graded according to the latest World Health Organization recommendations and the Nottingham histologic grading system, respectively.^1^, ^20^ All patients were female and Caucasian (median age at diagnosis, 60 years; range, 41–75 years; mean ± standard deviation, 61.0 ± 11.8 years). Pathologic re‐staging was performed following the 8th edition of the American Joint Committee on Cancer (AJCC) Cancer Staging Manual.^21^ Based on the quantity and quality of the available biomaterial (i.e., archival slides and blocks, residual extracted DNA), 12 cases were eligible for histochemistry and immunohistochemistry (IHC), 9 cases for fluorescent in situ hybridization (FISH), and 8 cases for next‐generation sequencing (NGS) profiling, as depicted in Figure S1.

### 2.2 Histochemistry and immunohistochemistry

Four‐micrometer‐thick sections were subjected to Alcian blue (pH 2.5) staining (to demonstrate the presence of intra‐ and extra‐cytoplasmatic mucopolysaccharides) and IHC using anti‐human antibodies against ER, PgR, Ki67, HER2, cytokeratin (CK)7, CK20, CK5/6, p40, p63, programmed death‐ligand 1 (PD‐L1, clone 22C3), EGFR, AREG, and mismatch repair proteins (MLH1, MSH2, MSH6, PMS2), as previously described.^22^ The IHC protocol uses two automated staining systems (i.e., Dako Omnis and Autostainer Link 48, Agilent) and anti‐human prediluted antibodies. For each antibody, positive and negative controls were included in each slide run. ER, PgR, and HER2 status were assessed according to the latest breast biomarker reporting guidelines published by the College of American Pathologists (CAP) in June 2021.^23^, ^24^ According to the updated recommendations from the International Ki67 Breast Cancer Working Group, a cut‐off value of ≥30% was used to define the high proliferation group.^25^ The epithelial component was highlighted by CK7 and CK20, while CK5/6, p40, and p63 were used to confirm the presence of basal/myoepithelial and/or epidermoid cells. PD‐L1 analysis was based on the combined positive score (CPS), determined as the number of PD‐L1+ tumor cells, lymphocytes, and macrophages divided by the total number of viable tumor cells, multiplied by 100.^26^ EGFR immunoreactivity was scored based on the membranous and/or cytoplasmic staining, as follows: 0, no staining or faint staining in ≤10% of tumor cells; 1+, weak staining in >10% of tumor cells; 2+, moderate staining in >10% of tumor cells; 3+, strong staining in ≥10% of tumor cells.^27^ The AREG expression was assessed by counting the percentage of positive cells (0%–100%) and multiplied by the staining intensity from 0 to 3+ with a total score ranging from 0 to 300. Tumors scored <200 were considered low‐expressors (AREG‐L); all the others were categorized as high‐expressors (AREG‐H).^27^ For each MMR protein, the loss of expression was defined by the complete absence of nuclear staining within all neoplastic cells.^28^ Cancers showing retained expression of MLH1, MSH2, MSH6, and PMS2 across the entire tumor were defined as MMR‐proficient (pMMR), irrespective of the staining intensity.^29^ In the presence of internal positive control (i.e., tumor microenvironment cells and non‐neoplastic epithelial cells from the terminal duct‐lobular unit), the complete loss of at least one of these proteins across the entire tumor designated the MMR‐deficient (dMMR) status.^30^ Low expression of at least one protein was classified as MMR‐low status.^31^ When the protein was expressed only in a part of the tumor and/or the immunoreactivity was faint compared to internal positive controls, the case was recorded as MMR‐heterogeneous/low (MMR‐h/low).^32^ The list of antibodies, clones, dilutions, antigen retrieval methods, and detailed scoring methods adopted are available in Table S1.

### 2.3 Tumor‐infiltrating lymphocytes (TILs) assessment

The evaluation of TILs was performed on 4‐μm‐thick hematoxylins and eosin (H&E)‐stained sections at a magnification of ×200, based on the recommendations by the International TILs Working Group.^33^ Specifically, TILs percentage was reported only for the stromal compartment (the area of stromal tissue occupied by mononuclear inflammatory cells over the total intratumoral stromal area). TILs outside of the tumor border and around ductal carcinoma in situ (DCIS) and normal terminal duct‐lobular units were excluded from the analysis. For the present study, the percentage of TILs was recorded as a continuous value and subcategorized as negative (<1%), low (1%–20%), intermediate (21%–50%), and high (>50%).

### 2.4 Fluorescent in situ hybridization (FISH)

Four‐micrometer‐thick sections from 11 samples underwent FISH to identify breaks in *MAML2* using ZytoLight® SPEC MAML2 dual‐color break‐apart probe (ZytoVision Ltd, Bremerhaven, Germany). This probe can detect MAML2 rearrangements irrespective of the fusion partner, including the *CRTC1‐MAML2* and *CRTC3‐MAML2* fusions.^34^ Similarly, the Vysis LSI EWSR1 (22q12) dual‐color break‐apart rearrangement probe kit was used for investigating *EWSR1* translocation. According to the manufacturer's protocols, the nuclei were counterstained with 4′,6‐diamidino‐2‐phenylindole (DAPI), and samples were evaluated by fluorescence microscopy Zeiss Axio Imager Z2 (Zeiss) combined with Metafer4‐MetaCyte system version V 3.14.143 (MetaSystems), as an acquisition system.^35^ Cells without t(11;19) (q21;p13) translocation show fused green and red signals, typically resulting in a yellow signal. A positive result was defined as the presence of a visible translocation (separation of red and green signals ≥2 signal diameters) in >10%–15% of the cells.

### 2.5 DNA extraction and next‐generation sequencing (NGS) analysis

Seven unstained slides at 4‐μm‐thick sections from representative formalin‐fixed paraffin‐embedded (FFPE) tissue blocks (*n* = 8) were used for the analyses. In seven of the eight cases (87.5%), manual microdissection was performed before nucleic acid isolation to enrich tumor cell content using a sterile scalpel. DNA was extracted using the Maxwell® RSC DNA FFPE Kit (Promega, Madison, WI, USA) following the manufacturer's instructions and then quantified by the QuantiFluor® ONE dsDNA System (Promega) on the Quantus™ Fluorometer (Promega). The mutational analyses were performed through the NGS panel Oncomine Comprehensive Assay (OCA) v3 System (ThermoFisher Scientific), which evaluates the mutational status (single‐nucleotide variants (SNV), insertions/deletions, and copy number variations (CNV)) of 161 cancer‐related and clinically actionable genes, as previously described.^36^ A full list of the genes included in this panel is available online (https://www.Thermofisher.com/order/catalog/product/A35805). Briefly, 10 ng of genomic DNA was used for the library preparation, and the subsequent Ion 540™ chips (ThermoFisher Scientific) loading was performed automatically on the Ion Chef™ System (ThermoFisher Scientific). Sequencing was performed using the Ion S5™ System (ThermoFisher Scientific) and data were analyzed using the Ion Reporter™ Software (v. 5.16) (ThermoFisher Scientific). Only mutations with an allele frequency ≥5% and with adequate quality metrics were reported. Mutations were classified as actionable/pathogenic based on the annotation in three different publicly available cancer genomics data sets (i.e., cBioPortal, https://www.cbioportal.org/,^37^, ^38^ ClinVar, https://www.ncbi.nlm.nih.gov/clinvar/, and Catalogue of Somatic Mutations in Cancer (COSMIC), https://cancer.sanger.ac.uk/cosmic). Clinically relevant and borderline alterations were visually inspected using the Integrative Genomics Viewer (IGV) software (Broad Institute and the Regents of the University of California). The median absolute pairwise difference (MAPD) metric was used to identify low‐quality samples at risk of generating false results and therefore needed to be excluded; only cases with MAPD of <0.5 were included.

### 2.6 Microsatellite instability (MSI) analysis

MSI status was evaluated in two patients using an automated Idylla MSI™ Test (Biocartis NV) targeting seven monomorphic homopolymer biomarkers (i.e., ACVR2A, BTBD7, DIDO1, MRE11, RYR3, SEC31A, and SULF2). At least five out of the seven biomarkers must be fully analyzed to consider the result of the test valid. To be considered as MSI, tumors should harbor alterations in >2 microsatellite loci.^39^

## 3 RESULTS

### 3.1 Histologic and immunohistochemical features of breast MEC

**FIGURE 1:**
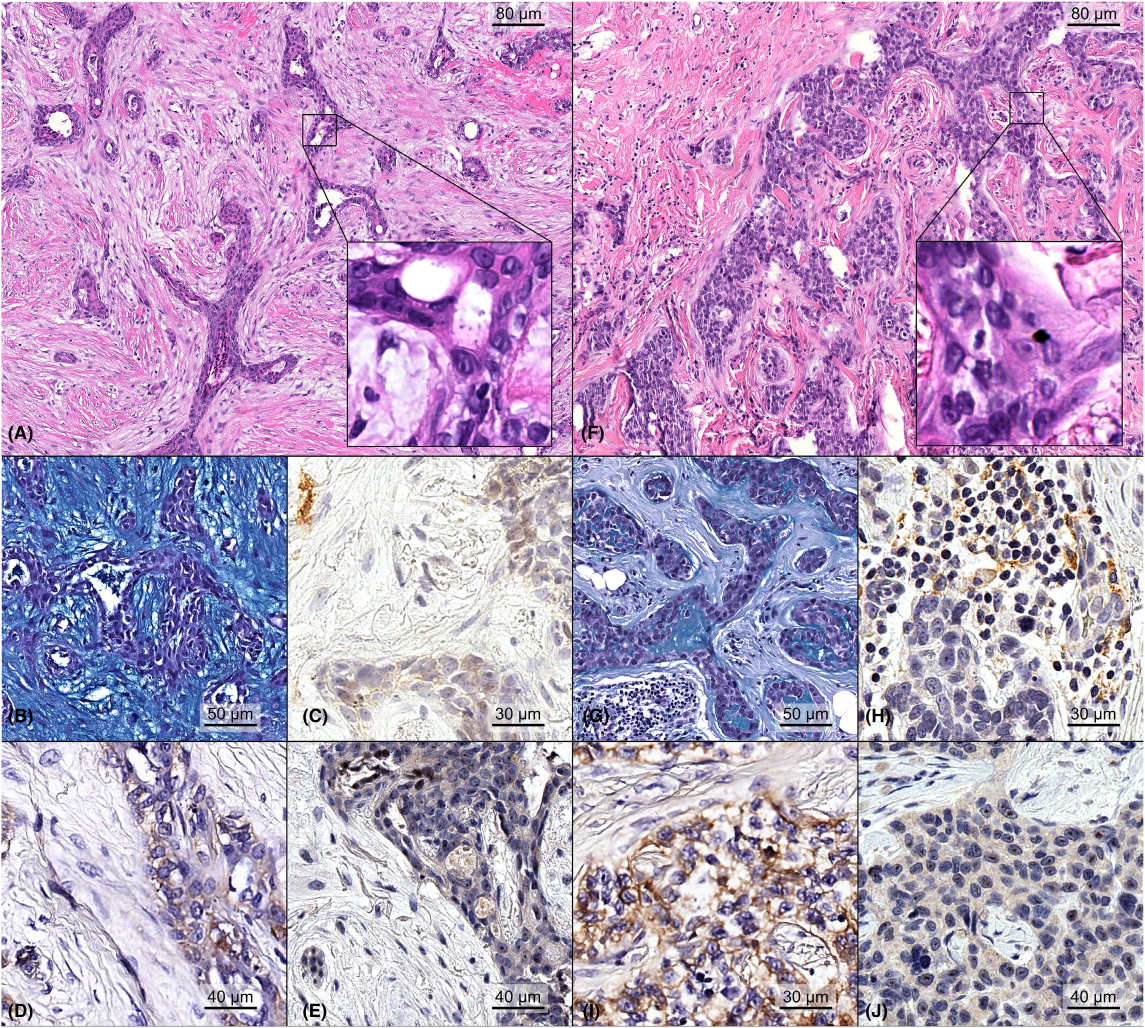
Representative micrograph showing the histopathological features of two primary breast mucoepidermoid carcinomas. Case #05 was a low‐grade carcinoma showing cystic ductal spaces lined by mucinous epithelial cells showing an unremarkable degree of nuclear pleomorphism and no mitotic count (A, H&E original magnification ×100; inset original magnification ×400), surrounded by a paucicellular myxoid stroma, as highlighted by Alcian blue stain (B, original magnification ×200). No PD‐L1 positivity was restricted to the neoplastic cells, with a CPS score of 10 (C, original magnification ×200). This neoplasm showed moderate cytoplasmic staining for EGFR in the majority of tumor cells and was scored as 2+ (D, original magnification ×200), while AREG expression was low (E, original magnification ×200). Case #3 was a high‐grade carcinoma showing nests of tumor cells with mucinous and squamoid features with no keratinization, minimal/null cystic formation, variable degree of nuclear atypia, occasional mitoses, karyopyknosis (F, H&E original magnification ×100; inset original magnification ×400), and diminished stromal mucin production in the presence of sparse mucin pools between the neoplastic clusters (G, original magnification ×200). The presence of TILs was confirmed by the expression of PD‐L1, with a CPS scored as 25 (H, original magnification ×200). This neoplasm was EGFR‐positive (I, original magnification ×200) and AREG low (J, original magnification ×200).

**FIGURE 2:**
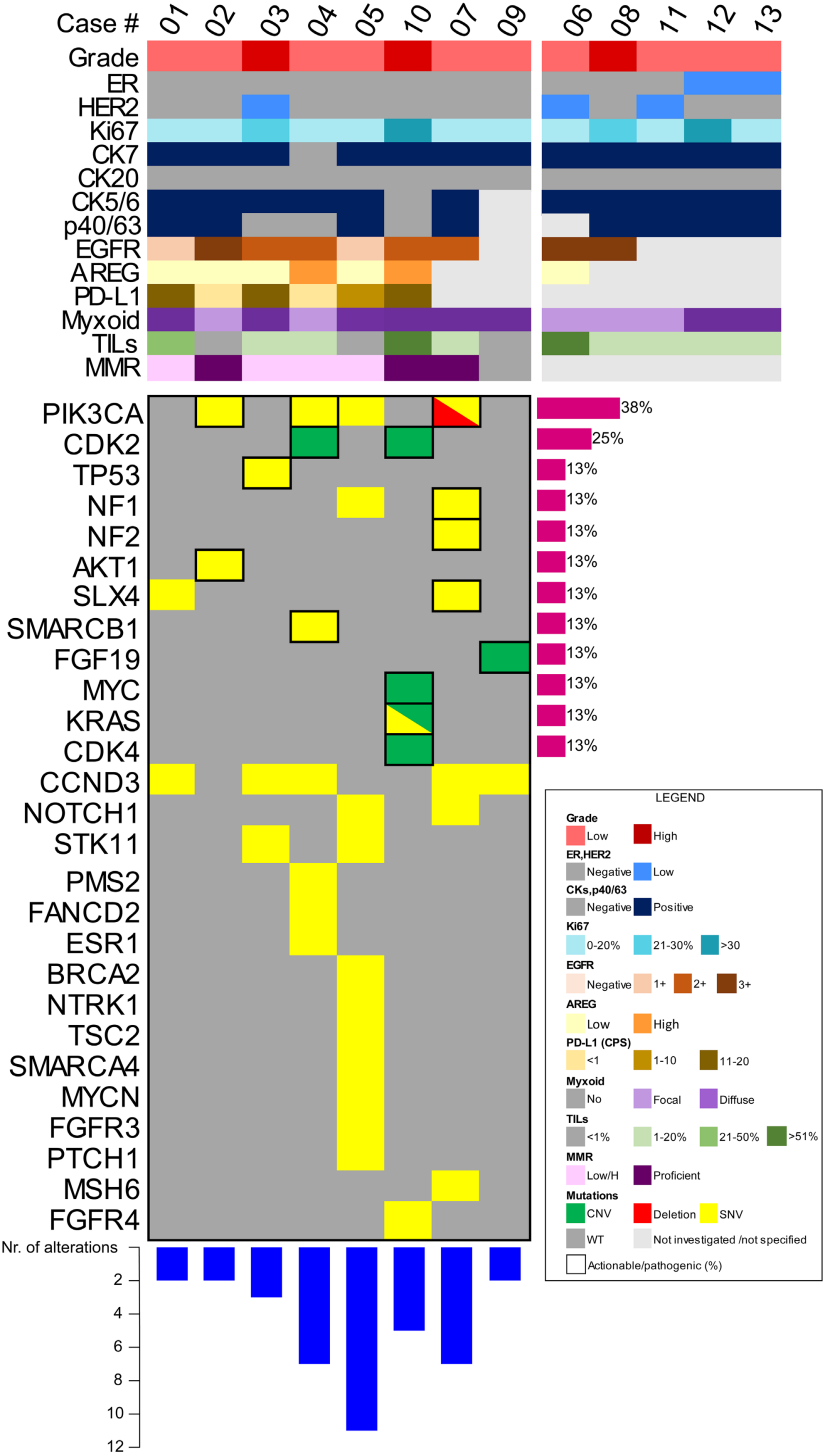
Heatmap illustrating selected clinicopathologic features, histochemical, and mutational status, including single‐nucleotide variants and copy‐number alterations, of MECs. Each column represents a patient, according to their ID, each row represents a clinicopathologic and genetic parameter, color‐coded according to the legend on the bottom right. The number of genomic alterations detected in each case is represented in the bar chart at the bottom of the figure, while the frequency of recurrent actionable/pathogenic mutations is reported on the right as a percentage. AREG, amphiregulin; CK, cytokeratin; CNV, copy‐number variations; CPS, combined positive score; ER, estrogen receptor; MMR, mismatch repair; PD‐L1, programmed death‐ligand 1; SNV, single‐nucleotide variants; TILs, tumor‐infiltrating lymphocytes; WT, wild‐type.

All MECs displayed ill‐formed glands featuring mucoid, epidermoid, and intermediate cells and variable degrees of cystic growth patterns within a desmoplastic/fibroid myxoid stroma (Figure 1). The majority of cases were of low histological grade and showed a low Ki67 labeling index (*n* = 10, 77%). The IHC analysis for epithelial and myoepithelial markers confirmed the presence of both types of cells in all tumors. When present, the cystic areas were lined by mucous cells, smaller eosinophilic cells, and solid components (mostly prevalent in high‐grade lesions) demonstrated basaloid cells gradually merged into epidermoid and mucous cells. All cases but one were Alcianophilic, showing intra‐ and extra‐cytoplasmic deposits of mucopolysaccharides (*n* = 12, 92%). Despite the presence of squamous differentiation in 4 (31%) cases, no keratinization was observed in all cases, excluding the possible differential diagnosis of breast adenosquamous carcinoma. At the histopathological revision, 2 (15%) cases showed very faint expression of ER in less than 10% of the neoplastic cells and were re‐classified as ER‐low. Furthermore, 3 (23%) HER2‐negative cases displayed a HER2‐low phenotype, that is, IHC score 1+ or 2+ with no gene amplification. The clinicopathologic characteristics of the patients included in this study are summarized in Table 1 and depicted at a single‐case level in Figure 2 and Table 2.

**TABLE 1:** Clinicopathological features of the patients included in this study.

	Patients (*n* = 13)
Age at diagnosis, range (median)	41–75 (60)
Metaplastic component, *n* (%)	
Yes	4 (31)
No	9 (69)
TNM descriptors, *n* (%)	
T	
T1a	1 (8)
T1b	3 (23)
T1c	4 (30)
T2	2 (15)
T3	1 (8)
T4b	1 (8)
n/a	1 (8)
N	
N0	11 (85)
N3	1 (8)
n/a	1 (8)
M	
M0	10 (77)
M1	2 (15)
n/a	1 (8)
LVI	
0	11 (85)
1	1 (8)
n/a	1 (8)
Grade, *n* (%)	
High	3 (23)
Low	10(77)
ER, *n* (%)	
Positive	0 (0)
Low	2 (15)
Negative	11 (85)
PgR, *n* (%)	
Positive	0 (0)
Negative	13 (100)
HER2, *n* (%)	
Positive	0 (0)
Low	3 (23)
Negative	10 (77)
Ki67, *n* (%)	
High	3 (23)
Low	10 (77)
Molecular subtype, *n* (%)	
Luminal A like	0 (0)
Luminal B like	2 (15)
HER2‐type	0 (0)
TNBC	11 (85)
PD‐L1, *n* (%)	
≥10	4 (67)
<10	2 (33)
TILs, *n* (%)	
Absent	3 (25)
Low	6 (50)
Intermediate	1(8)
High	2 (17)
MMR, *n* (%)	
pMMR	2 (33)
MMR‐h/low	5 (67)
dMMR	0 (0)

Abbreviations: d, deficient; ER, estrogen receptor; h, heterogeneous; low, low; LVI, lymphovascular invasion; MMR, mismatch repair; p, proficient; PD‐L1, programmed death‐ligand 1; PgR, progesterone receptor; TILs, tumor infiltrating lymphocytes; TNBC, triple‐negative breast cancer.

**TABLE 2:** Histochemical and immunohistochemical characteristics of breast MECs.

ID	CK7	CK20	CK5/6	p40/p63	EGFR	AREG	TILs	PD‐L1	ALCIAN BLUE	MLH1	MSH2	MSH6	PMS2
MUC_001	+	−	+	+	1+	low (180)	35%	20	3	R	R	H	H
MUC_002	+	−	+	+	3+	low^20^	0%	0	1	R	R	R	R
MUC_003	+	−	+	−	2+	low (80)	5%	15	2	R	R	R	L
MUC_004	−	−	+	−	2+	high (240)	20%	0	1	R	R	H	R
MUC_005	+	−	+	+	1+	low (120)	0%	10	3	H	R	H	H
MUC_006	n/a	n/a	n/a	n/a	3+	low (40)	80%	n/a	1	n/a	n/a	n/a	n/a
MUC_007	n/a	n/a	n/a	+	2+	n/a	2%	n/a	2	n/a	n/a	n/a	n/a
MUC_008	n/a	n/a	+	+	3+	n/a	5%	n/a	1	n/a	n/a	n/a	n/a
MUC_009	n/a	n/a	n/a	n/a	n/a	n/a	n/a	n/a	n/a	n/a	n/a	n/a	n/a
MUC_010	+	−	−	−	2+	high (300)	80%	20	2	R	R	R	R
MUC_011	n/a	n/a	n/a	n/a	n/a	n/a	10%	n/a	1	n/a	n/a	n/a	n/a
MUC_012	n/a	n/a	n/a	n/a	n/a	n/a	2%	n/a	3	n/a	n/a	n/a	n/a
MUC_013	n/a	n/a	n/a	+	n/a	n/a	0%	n/a	2	n/a	n/a	n/a	n/a

Abbreviations: AREG, amphiregulin; CK, cytokeratin; H, heterogeneous; L, low; MLH1, mutL homolog 1; MSH2, mutS homolog 2; MSH6, mutS homolog 6; PD‐L1, programmed death ligand 1; PMS2, postmeiotic segregation increased 2; R, retained; TILs, Tumor‐infiltrating lymphocytes.

### 3.2 Lack of *MAML2* and/or *EWSR1* rearrangements in breast MEC

To define whether primary breast MECs harbor the hallmark diagnostic biomarker of salivary MECs, we investigated the presence of *MAML2 and/or EWSR1* fusions by FISH. No gene rearrangements were detected in the tested cases (*n* = 9), as shown in Figure S2 and detailed in Table S2.

### 3.3 Overexpression of the EGFR/AREG axis in breast MEC

Next, we asked whether the EGFR/AREG axis is activated in breast MEC, even in the absence of *MAML2* rearrangements. All cases with enough material for additional ancillary studies (*n* = 9) showed EGFR overexpression. Among these, 3 (33%), 4 (44%), and 2 (22%) cases were scored by IHC as 3+, 2+, and 1+, respectively. Furthermore, all cases were AREG‐positive (AREG‐L *n* = 5/7, 72% and AREG‐H *n* = 2/7, 28%) (Figure 2). These results suggest that increased EGFR and AREG expression in breast MECs can occur even in fusion‐negative cases.

### 3.4 Low TILs and PD‐L1 levels in breast MEC

Then, we assessed the expression of immune‐related markers considering their potential prognostic and predictive value in TNBCs, as detailed in Table 1 and Table 2. TILs were quantified in 12 of 13 tumors (92%), among these, only 3 of the 12 (25%) had no TILs. Among the six cases with available material for additional IHC analyses, four (67%) MECs had PD‐L1 CPS ≥ 10. These results suggest an activation of the antitumor immune response in breast MECs.

### 3.5 Recurrent mutations in cancer genes and PI3K/AKT/mTOR and cell cycle regulation pathways in breast MECs

**FIGURE 3:**
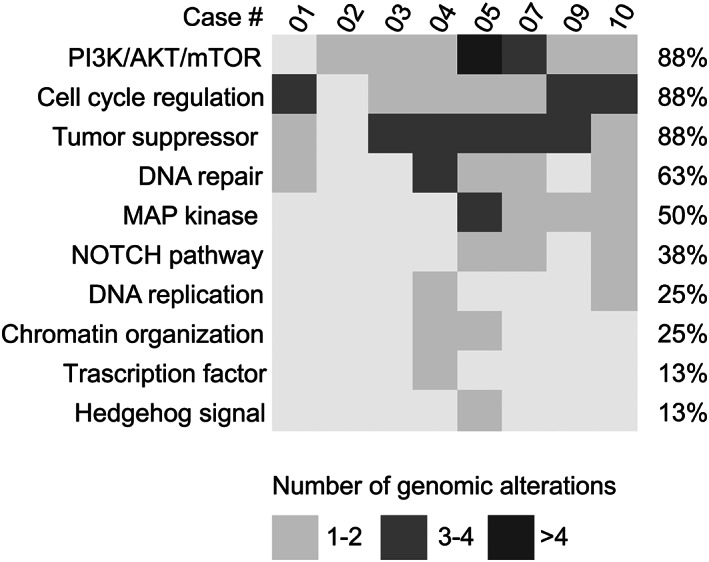
Recurrently altered pathways in breast MECs. Each column represents a patient, each row a pathway; the number of molecular aberrations is annotated as reported on the bottom.

Taken together, 8 of the13 (62%) cases had sufficient DNA quantity and/or quality for molecular profiling through a comprehensive NGS panel targeting 161 cancer‐related and clinically actionable genes. All cases harbored at least 2 molecular alterations (range, 2–11; median number of mutations, 4), as shown in the heatmap presented in Figure 2. The cases with a higher mutational burden (#4, #5, #7, and #10) were enriched for alterations affecting the phosphoinositide 3 kinase (PI3K)/Akt/mammalian target of rapamycin (mTOR), cell cycle regulation, and DNA repair pathways. In particular, *PIK3CA* was the most frequently altered gene in the whole cohort of patients, with mutations observed in 4 (50%) cases which included the recurrent hotspot mutations p.H1047R, p.M1043I, and p.T1025I (Table S3). Additional somatic mutations affecting cancer‐ genes found in MEC included *NF1/2*, structure‐specific endonuclease subunit (*SLX4*), and Notch homolog 1, and translocation‐associated (*NOTCH1*) in 2 (25%) cases as well as *AKT1*, SWI/SNF‐related matrix‐associated actin‐dependent regulator of chromatin subfamily B member 1 (*SMARCB1*), *KRAS*, and *BRCA2* in 1 (13%) case, respectively. A pathogenic *TP53* mutation (p.C238Y) was observed in one case (#3), which was a high‐grade MEC with HER2‐low expression; this mutation was mutually exclusive with PIK3CA. The CNVs analysis revealed gains in well‐known cancer genes, such as cyclin‐dependent kinase (*CDK*)*2*, *CDK4*, fibroblast growth factor 19 (*FGF19*), *MYC*, and *KRAS*. All the genetic alterations along with the clinicopathological features of the cases included in this study are detailed in Figure 2 and summarized in Table S3. Considering both SNVs and CNAs, enrichment was observed in the PI3K/AKT/mTOR and cell cycle regulation pathways (Figure 3). Regarding the former, the altered genes included *AKT1*, *PIK3CA*, serine/threonine kinase 11 (*STK11*), *NF1*, tuberous sclerosis complex 2 (*TSC2*), and *FGF3/4*, with mutation detected in the vast majority (*n* = 7, 88%) of cases. The most frequently affected genes of the cell cycle regulation pathways identified in 88% of cases were *CDK2*, *CDK4*, cyclin D3 (*CCND3*), *STK11*, and *MYC*. Similarly, tumor suppressor genes, including *TP53*, *STK11*, *BRCA2*, and *NF2* were also mutated in 88% of the cases analyzed. Additionally, a relatively high proportion of cases (*n* = 5/8, 63%) harbored mutations in genes involved in DNA repair pathways (e.g., *SLX4*, *BRCA2*, *FANCD2*, *PMS2*, *MSH6*). None of the cases, including those that harbored mutations in MMR genes, showed aberrations in the MMR system when assessed by IHC (Table S4). Cases with mutations in MMR genes did not exhibit MSI.

## 4 DISCUSSION

Here, we provide the most comprehensive molecular landscape of the largest collection of breast MECs available in the literature to date, delivering new data on this vanishingly rare and potentially underdiagnosed TNBC subtype. Our analyses confirm that not all breast MECs are low‐grade TNBCs suggesting that they should undergo a thorough multidisciplinary discussion for appropriate clinical management. Furthermore, we provide previously unavailable evidence that the EGFR/AREG axis can be activated in breast MEC in the absence of the oncogenic rearrangements occurring in *MAML2* and/or *EWSR1*. This observation confirms on one hand that these rearrangements are not reliable diagnostic biomarkers for breast MECs (unlike those arising in salivary glands) and on the other hand it suggests that EGFR and AREG overexpression are not strictly dependent on such genetic changes. In addition, we investigated for the first time the tumor immune microenvironment features of breast MECs, showing TILs and PD‐L1 activation. Finally, we highlight recurrent somatic genetic alterations in cancer genes and highly oncogenic signaling pathways, positing that at least a subset of breast MECs can be more aggressive than other salivary gland‐like TNBCs.

As a group of tumors, TNBC consists of different histologic subtypes, with highly heterogeneous clinical behavior.^40^ Although breast MEC falls into the spectrum of TNBC, a thorough revision of our cases revealed 15% of ER‐low and 23% of HER2‐low cases, a feature that may gain momentum due to the availability of novel antibody‐drug conjugate drugs for metastatic TNBC.^41^, ^42^, ^43^, ^44^ In this context, our observation suggests that conventional biomarkers should be carefully tested in breast MEC not only for the different risk profiles but also for the potential therapeutic benefits in high‐risk individuals. In addition, consistent with previous reports,^45^ our series included 3 (23%) patients with MEC of high histological grade. Among these, distant metastases in sacral bone and lung were observed in one woman, a feature consistent with high‐risk TNBC natural history.^46^, ^47^ Furthermore, we also report a patient with a low‐grade tumor who developed distant metastasis, pointing to the diagnostic challenges provided by breast MEC.

Histologically, breast MECs display extensive morphological similarities with those arising in the salivary glands.^2^ Therefore, recent studies sought to determine whether the former would be underpinned by the molecular alterations that are reported to occur in their salivary gland counterparts.^48^ Interestingly, the t (11, 19)(q14–21; p12–13) translocation, which is the most frequent molecular alteration occurring in salivary gland MECs and results in the oncogenic *MAML2* fusions, has been found only in three cases of breast MEC.^18^, ^19^ It has been reported that such structural rearrangements can drive the EGFR/AREG axis activation, which is also a drive event in TNBC.^49^ In line with previous observations, no structural rearrangements were found in our study cohort but we observed that both EGFR and AREG were upregulated in all cases. It is known that high‐grade fusion‐negative salivary MECs are associated with multiple genomic imbalances and an unfavorable clinical outcome, while *MAML2* fusion‐negative and *EWSR1* fusion‐positive salivary MECs carry a slightly better prognosis.^16^, ^17^ Of note, activating *PIK3CA* mutations have been suggested to activate an EGFR/extracellular signal‐regulated kinase (ERK) paracrine signaling axis in TNBC.^50^ Our data support the contention that gene fusions might not be the only EGFR/AREG axis‐related activating mechanisms in breast MECs and that the absence of this alteration should not be used to exclude a possible diagnosis of MEC in the breast. Rather, a thorough histomorphological analysis should be carried out to confirm the pathognomonic features of MECs, including the absence of keratinization in the squamoid component, which is characteristic of adenosquamous breast cancer.^4^

The role of the tumor immune microenvironment has not been previously explored in breast MECs. To bridge this gap of knowledge, we characterized and quantified the presence of stromal TILs and tested the PD‐L1 and MMR status. Taken together, our cases were largely characterized by the presence of a different amount of TILs, suggesting an immune activation and possible tumor suppressor mechanisms.^51^ On the other hand, we documented a subset of PD‐L1+ MECs with CPS ≥ 10, implying a window of opportunity for immunotherapy combination strategies.^52^

Comprehensive molecular studies of TNBC have demonstrated a heterogeneous mutational landscape with frequent genetic alterations in *TP53* and *PIK3CA*.^53^ However, breast MECs have generally not been included in these studies. We found *PIK3CA* to be the most frequently altered gene showing highly recurrent pathogenic mutations in hotspot regions, at variance with a recent report failing to detect *TP53* or *PIK3CA* mutations in two breast MECs.^18^ Previous studies on salivary gland MEC reported alterations in *PIK3CA* occurring in intermediate‐ or high‐grade MEC but only exceptionally in low‐grade tumors. In both studies, *TP53* was the most commonly mutated gene and increased in frequency with increasing MEC grade.^54^ Alas, most publications on breast MEC do not provide significant information on genetic testing performed, *PIK3CA* alterations are widely detected in the invasive ductal carcinoma population and its triple‐negative subtypes.^4^, ^19^, ^54^ These mutations, specifically, have been also attributed as a common feature of breast adenomyoepitheliomas, which share a common histological component with breast MECs.^48^ It should be noted, however, that similar to our study, no *TP53* mutations were detected in pulmonary MEC, thereby demonstrating that *TP53* variations can differ between salivary gland MEC and MEC at other sites.^55^ Taken together, we observed the lack of hallmark *TP53* mutations typically found in high‐grade forms of TNBC and salivary gland MECs but the presence of pathogenic *PIK3CA* alterations, which in combination with EGFR overexpression could provide the evidence for a potential mechanism of tumorigenesis in breast MEC.

Our pathways analysis showed that along with mutations in *PIK3CA*, in most of the cases analyzed, genetic alterations were also detected in other genes of the PI3K/AKT/mTOR pathway suggesting its improper activation in breast MEC and its potential targetability with recently developed drugs.^56^ These findings are consistent with other studies where activating mutations in this pathway were more commonly observed in high‐grade than in low‐grade tumors.^54^ In addition, we detected enrichment of somatic genetic alterations in genes implicated in cell cycle regulation and DNA repair pathways. This is in agreement with previous findings both in salivary and pulmonary MECs.^54^, ^55^, ^57^ Mutations in cell cycle regulatory genes can result in the dysfunctionality of the cell cycle checkpoints and the inappropriate progress of the cell cycle leading to genomic instability.^58^ In our cohort, among the DNA repair genes, mutations were not only found in *BRCA* but also in MMR genes. However, our MMR immunohistochemical analyses, where only one case was found as MMR‐low, were in line with previous reports in TNBC that documented extremely low incidence rates.^59^ Furthermore, we observed that although *MMR* genes might be mutated, this is not always reflected by MMR deficiency in IHC and/or MSI.^28^, ^32^ Nevertheless, these findings raise the possibility of novel therapeutic targets for breast MECs since alterations in these genes can be targeted with PARP inhibitors and immunotherapy, respectively.^60^ Mutations in these genes, however, were annotated as of unknown significance in the publicly available datasets; thus, any postulated correlation between such genetic alterations and breast MEC development is still speculative, and worth to be explored in future studies.

Considering the rarity of MECs arising in the breast, no widely adopted guidelines are currently available for their clinical management. However, the vast majority of cases reported in the literature have been diagnosed at early stages. In these patients, if high‐risk features are detected (e.g., large tumor size, high histologic grade, high Ki67 index, lymph‐vascular invasion) surgery with sentinel lymph node are still recommended.^61^, ^62^, ^63^ On the other hand, MECs without any of the aforementioned characteristics, breast‐conserving surgery with clear margins would suffice to avoid overtreatment.^64^, ^65^, ^66^ In both scenarios, it is wise to adopt tailored follow‐up procedures.^67^, ^68^, ^69^ Similarly, the de‐escalation of adjuvant medical treatment, including chemotherapy, immunotherapy, targeted therapies, and radiotherapy requires a multidisciplinary approach.^70^, ^71^, ^72^ Hence, the paucity of comprehensive molecular information on large cohorts of patients prevents any specific recommendation and thus, our results should be validated and discussed in the context of multi‐institutional studies and dedicated cancer registries.

Our study has some limitations. First, given the rarity of MECs arising in the breast, we could analyze a relatively small number of cases, still representing the largest cohort of breast MECs in the literature. Second, considering the limited availability of material, not all the cases were fully analyzed. Third, survival analyses have not been performed, due to the small series of patients investigated. Large prospective studies are needed to assess the clinical outcome of patients with mammary MECs as well as the potential prognostic and predictive value of the biomarkers that are found altered in these tumors. Finally, given the small sample size, we were not able to extend the analysis to immune gene expression profiles through the construction of their immune landscape. This study, however, should be considered hypothesis‐generating and additional multicentric studies, coupled with comprehensive immune analysis would be required to identify MECs immune signatures.

In conclusion, breast MECs lack the hallmark TP53 typically found in high‐grade subtypes of TNBC and *MAML2* or *EWSR1* rearrangements found in salivary MECs. The immune milieu compared to TNBCs of no special type suggest an immunoediting activation. Finally, the EGFR/AREG axis activation, coupled with the complex patterns of mutations in PI3K/AKT/mTOR and cell cycle regulation pathways militate against the widely accepted belief that MECs should be invariably considered low‐risk TNBCs.

## AUTHOR CONTRIBUTIONS

**Konstantinos Venetis:** Data curation (lead); formal analysis (equal); investigation (equal); methodology (equal); software (equal); validation (lead); visualization (equal); writing – original draft (equal); writing – review and editing (equal). **Elham Sajjadi:** Data curation (lead); formal analysis (equal); investigation (equal); methodology (equal); project administration (lead); validation (equal); visualization (equal); writing – review and editing (equal). **Mariia Ivanova:** Formal analysis (equal); investigation (equal); methodology (equal); project administration (equal); visualization (equal); writing – review and editing (equal). **Silvia Andaloro:** Data curation (equal); investigation (equal); writing – original draft (equal). **Simona Pessina:** Formal analysis (equal); visualization (equal). **Chiara Zanetti:** Formal analysis (equal); visualization (equal). **Alberto Ranghiero:** Data curation (equal). **Gabriele Citelli:** Investigation (equal). **Chiara Rossi:** Formal analysis (equal); investigation (equal). **Marco Lucioni:** Formal analysis (equal); investigation (equal); resources (equal); supervision (equal). **Umberto Malapelle:** Methodology (equal); supervision (equal); writing – review and editing (equal). **Fabio Pagni:** Supervision (equal); validation (equal); writing – review and editing (equal). **Massimo Barberis:** Resources (equal). **Elena Guerini‐Rocco:** Funding acquisition (equal); methodology (equal); supervision (equal); validation (equal); visualization (equal); writing – review and editing (equal). **Giuseppe Viale:** Formal analysis (equal); methodology (equal); resources (equal); supervision (equal); writing – review and editing (equal). **Nicola Fusco**: Conceptualization, Funding acquisition (equal); methodology (equal); supervision (equal); validation (equal); visualization (equal); resources (equal); writing – review and editing (equal).

## FUNDING INFORMATION

This research was funded by the Italian Ministry of Health with Ricerca Corrente funds.

## CONFLICT OF INTEREST STATEMENT

U.M. has received personal fees (as a consultant and/or speaker bureau) from Boehringer Ingelheim, Roche, MSD, Amgen, Thermo Fisher Scientifics, Eli Lilly, Diaceutics, Jannsen, Diatech, Hedera, GSK, Merck and AstraZeneca. M.B. from MSD Oncology, Roche/Genetech, Astra Zeneca, Thermofisher Scientific, and Illumina. E.G.R. from Thermo Fisher Scientific, Novartis, AstraZeneca, Roche, Biocartis, and Illumina. G.V. from MSD Oncology, Pfizer, Dako, Roche/Genetech, Astellas Pharma, Novartis, Bayer, Daiichi; Sankyo, Menarini, Ventana Medical Systems Dako/Agilent Technologies, Cepheid, and Celgene. N.F. from Merck Sharp & Dohme (MSD), Boehringer Ingelheim, Novartis, AstraZeneca, and Daiichi‐Sankyo. These companies had no role in the design of the study; in the collection, analyses, or interpretation of data; in the writing of the manuscript, and/or in the decision to publish the results. All other authors declare no potential conflicts of interest.

## ETHICS APPROVAL/CONSENT TO PARTICIPATE

This study is in line with the local ethical guidelines and was approved by the local Ethical Committee under protocol number #UID3472.

## Supporting information


Figure S1.

Figure S2.

Table S1.
Table S2.
Table S3.
Table S4.

## Data Availability

The datasets used and/or analyzed during the current study are publicly available at https://www.ncbi.nlm.nih.gov/sra under the BioProject ID: PRJNA900207.
