# Supplementary figures and images for: The molecular landscape of breast mucoepidermoid carcinoma
Source: Cancer Med. 2023 Mar 14;12(9):10725–37. doi: 10.1002/cam4.5754 (PMC10225218; doi:10.1002/cam4.5754)

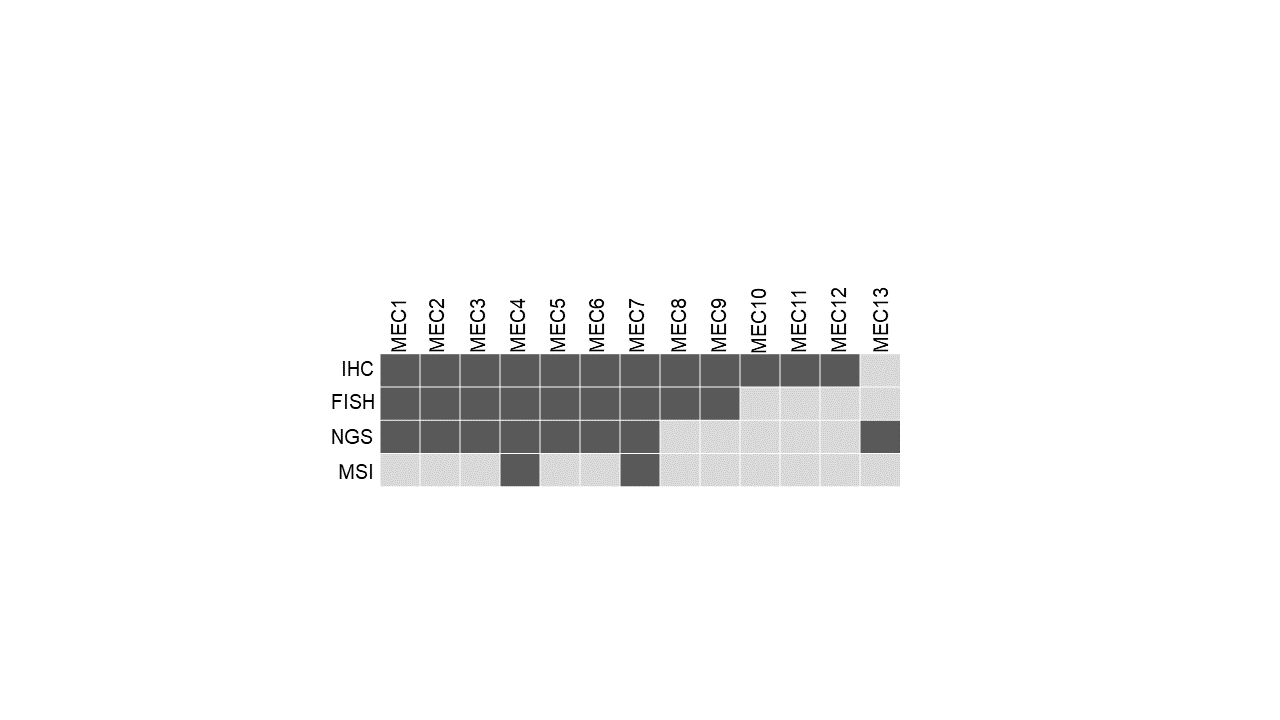

Supplement: Supplementary file 1 — Figure S1. [file CAM4-12-10725-s003.PNG]

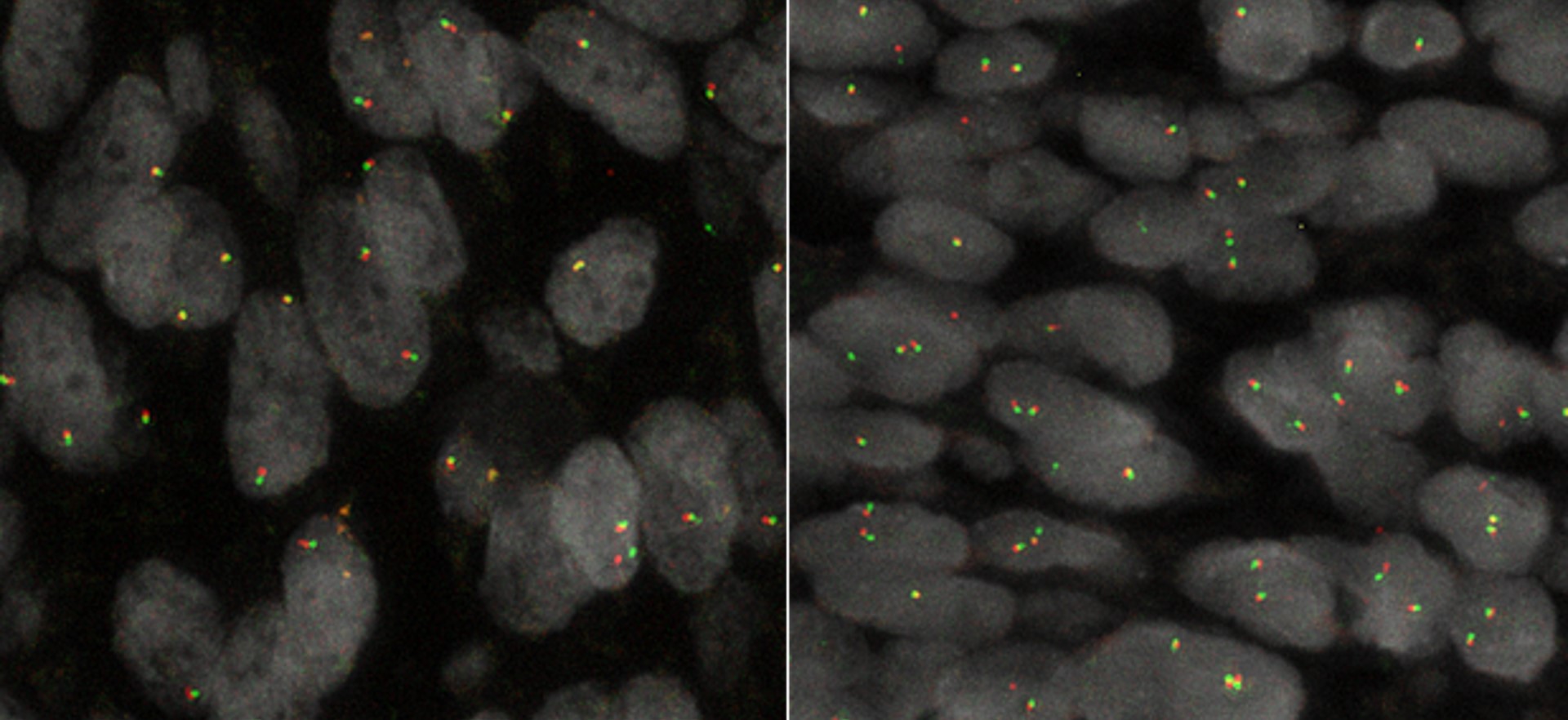

Supplement: Supplementary file 2 — Figure S2. [file CAM4-12-10725-s001.jpg]
