# Supplementary material for: The molecular landscape of breast mucoepidermoid carcinoma
Source: Cancer Med. 2023 Mar 14;12(9):10725–37. doi: 10.1002/cam4.5754 (PMC10225218; doi:10.1002/cam4.5754)
Supplement: Supplementary file 3 — Table S1. Table S2. Table S3. Table S4. [file CAM4-12-10725-s002.docx]

| **Marker** | **Clone** | **Dilution** | **Technology** | **Antigen retrieval** | **Scoring** |
| --- | --- | --- | --- | --- | --- |
| ER | EP1 | Ready to use | Omnis | EnVision FLEX, Low pH 20’ | ASCO/CAP and St Gallen guidelines; positive if ≥1% of tumor cell nuclei are immunoreactive. |
| PgR | PgR 636 | 1:100 | Omnis | EnVision FLEX, Low pH 30’ | ASCO/CAP and St Gallen guidelines; positive if ≥1% of tumor cell nuclei are immunoreactive. |
| HER2 | Polyclonal | 1:400 | Omnis | EnVision FLEX, Low pH 30’ | ASCO/CAP guidelines; 3+ if complete membrane staining that is intense and >10% of tumor cells; 2+ if weak to moderate complete membrane staining in >10% of tumor cells or complete membrane staining that is intense but within ≤10% of tumor cells; 1+ if incomplete membrane staining that is faint/barely perceptible and within >10% of tumor cells; 0 if no staining observed or membrane stating that is incomplete and is faint/barely perceptible and within ≤10% of tumor cells. |
| Ki67 | MIB1 | Ready to use | Omnis | EnVision FLEX, Low pH 30’ | International Ki67 in Breast Cancer Working Group; high if ≥30% of tumor cell nuclei are immunoreactive. |
| CD68 | PG-M1 | 1:100 | Omnis | EnVision FLEX, Low pH 30’ | Positive for any intensity of membrane/cytoplasm staining; high if ≥26 immunoreactive cells. |
| CK7 | OV-TL 12/30 | 1:400 | Omnis | EDTA pH 9.0 | Positive for any intensity of membrane/cytoplasm staining (normal breast tissue, mucoid cells) |
| CK20 | Ks20.8 | 1:80 | Omnis | EDTA pH 9.0 | Positive for any intensity of membrane/cytoplasm staining (primary mucinous tumors) |
| CK5/6 | D5/16B4 | 1:50 | Omnis | EDTA pH 9.0 | Positive for any intensity of cytoplasm staining (basal and myoepithelial cells) |
| p40 | BC28 | 1:100 | Omnis | Citrate pH 6.0 | Positive for >5% of nuclear staining (myoepithelial cells of breast) |
| p63 | DAK-P63 | 1:50 | Omnis | Citrate pH 6.0 | Positive for >5% of nuclear staining (myoepithelial cells of breast) |
| PD-L1 | 22C3 | Ready to use | Autostainer | Citrate pH 6.0 | Combined positive score (CPS); number of PD-L1 positive tumor cells, lymphocytes, and macrophages divided by the total number of viable tumor cells, multiplied by 100. |
| EGFR | 31G7 | 1:20 | Omnis | ProteinaseK | 0, no staining or faint staining in ≤ 10% of tumor cells; 1+, weak staining in > 10% of tumor cells; 2+, moderate staining in > 10% of tumor cells; 3+, strong staining in ≥ 10% of tumor cells. |
| AREG | IgG-Anti goat | 1:50 | Autostainer | EDTA pH 9.0 | Positive membrane/cytoplasm staining (intensity, 0-3+) x % positive cells. Final score 0-300. |
| **MMR**  MLH1  MSH2  MSH6  PMS2 | ES05  FE11  EP49  EP51 | Ready to use  Ready to use  Ready to use  Ready to use | Omnis  Omnis  Omnis  Omnis | EDTA pH 9.0  EDTA pH 9.0  EDTA pH 9.0  EDTA pH 9.0 | MMR-proficient (pMMR), in case of retained expression, irrespective of the staining intensity  MMR-heterogeneous (hMMR), the protein was expressed only in a part of the tumor (i.e., <100% of tumor cells) in the presence of positive internal control  MMR-low, low expression of at least one of the proteins in the presence of positive internal control  MMR-deficient (dMMR), in case of complete loss of at least one of proteins in the presence of positive internal control. |
| **Supplementary Table S1.**  List of antibodies, clones, dilutions, antigen retrieval methods, and scoring systems adopted for immunohistochemical analyses. ER, estrogen receptor alpha; PgR, progesterone receptor; EFGR, epidermal growth factor receptor; AREG, amphiregulin. | | | | | |

| **ID** | **MAML2 break** | **EWSR1 break** |
| --- | --- | --- |
| MUC_001 | absent | absent |
| MUC_002 | absent | absent |
| MUC_003 | absent | Absent |
| MUC_004 | absent | absent |
| MUC_005 | absent | absent |
| MUC_006 | absent | absent |
| MUC_007 | absent | absent |
| MUC_008 | absent | n/a |
| MUC_009 | n/a | n/a |
| MUC_010 | absent | absent |
| MUC_011 | n/a | n/a |
| MUC_013 | n/a | n/a |
| **Supplementary Table S2.** Study cohort FISH results (MAML2 break, EWSR1 break) | | |

| **Patient ID** | **Gene** | **Amino acid change** | **Coding** | **MAF (%)** | **Coverage** | **CNV** | |
| --- | --- | --- | --- | --- | --- | --- | --- |
| #01 | CCND3 | p.S259A | c.775 T>G | 49 | 1736 | - | |
|  | SLX4 | p.V894G | c.2681 T>G | 53 | 1024 | - | |
|  | SLX4 | p.M386V | c.1156 A>G | 44 | 1957 | - | |
| #02 | AKT1 | p.D323N | c.967 G>A | 12 | 301 | - | |
|  | PIK3CA | p.H1047R | c.3140 A>G | 5 | 1666 | - | |
| #03 | STK11 | p.R425H | c.1274 G>A | 10 | 134 | - | |
|  | TP53 | p.C238Y | c.713 T>G | 24 | 1399 | - | |
|  | CCND3 | p.S259A | c.775 T>G | 40 | 1035 | - | |
| #04 | PIK3CA | p.H1047R | c.3140 A>G | 39 | 2000 | - | |
|  | PMS2 | p.T499I | c.1496 C>T | 20 | 168 | - | |
|  | FANCD2 | p.I935L | c.2803 A>C | 50 | 596 | - | |
|  | SMARCB1 | p.R377H | c.1130 G>A | 20 | 154 | - | |
|  | CCND3 | p.S259A | c.775 T>G | 40 | 312 | - | |
|  | ESR1 | p.V392I | c.1174 G>A | 47 | 2000 | - | |
|  | CDK2 | - | - | - |  | 6.94 | |
| #05 | PIK3CA | p.M1043I | c.3129 G>A | 5 | 1947 | - | |
|  | STK11 | p.D358N | c.1072 G>A | 26 | 232 | - | |
|  | NF1 | p.R1132C | c.3394 C>T | 58 | 1287 | - | |
|  | BRCA2 | p.D237N | c.709 G>A | 65 | 1850 | - | |
|  | NOTCH1 | p.V413M | c.1237 G>A | 32 | 129 | - | |
|  | NTRK1 | p.P335L | c.1004 C>T | 63 | 815 | - | |
|  | TSC2 | p.E1756K | c.5266 G>A | 29 | 138 | - | |
|  | SMARCA4 | p.Q118* | c.352 C>T | 23 | 219 | - | |
|  | SMARCA4 | p.T1032I | c.3095 C>T | 20 | 349 | - | |
|  | MYC | p.V78I | c.232 G>A | 26 | 179 | - | |
|  | FGFR3 | p.A15T | c.43 G>A | 29 | 121 | - | |
|  | PTCH1 | p.P1295L | c.3884 C>T | 23 | 195 | - | |
| #07 | PIK3CA | p.T1025I | c.3074 C>T | 17 | 537 | - | |
|  | PIK3CA | p.G106_E109del | c.317_328del | 32 | 1957 | - | |
|  | NF1 | p.W696* | c.2087 G>A | 49 | 124 | - | |
|  | NF2 | p.Q298* | c.892 C>T | 22 | 426 | - | |
|  | MSH6 | p.D390N | c.1168 G>A | 20 | 385 | - | |
|  | CCND3 | p.S259A | c.775 T>G | 21 | 260 | - | |
|  | NOTHC1 | p.D1870N | c.5608 G>A | 22 | 277 | - | |
|  | SLX4 | p.D1158N | c.3472 G>A | 20 | 416 | - | |
| #09 | CCND3 | p.S259A | c.775 T>G | 51 | 1906 | - | |
|  | FGF19 | - | - | - | - | 6.36 | |
| #10 | KRAS | p.G12C | c.34 G>T | 72 | 1999 | - | |
|  | BRCA2 | p.R2336C | c.7006 C>T | 70 | 1981 | - | |
|  | FGFR4 | p.V10I | c.28 G>A | 68 | 846 | - | |
|  | MYC | - | - | - | - | 8.06 | |
|  | KRAS | - | - | - | - | 7.96 | |
|  | CDK2 | - | - | - | - | 7.28 | |
|  | CDK4 | - | - | - | - | 7.70 | |
| **Supplementary Table S3.** Overview of genetic alterations identified in 8 cases analyzed by next generation sequencing (NGS). MAF, mutant allele frequency, CNV, copy number variation. | | | | | | |  |

| **ID** | **MLH1** | **MSH2** | **MSH6** | **PMS2** |  |
| --- | --- | --- | --- | --- | --- |
| MUC_001 | R | R | H | H |  |
| MUC_002 | R | R | R | R |  |
| MUC_003 | R | R | R | L |  |
| MUC_004 | R | R | H | R |  |
| MUC_005 | H | R | H | H |  |
| MUC_010 | R | R | R | R |  |
| **Supplementary Table S4.** Mismatch repair proteins expression assessment. R, retained expression; H, heterogenous expression; pMMR, MMR-proficient status; dMMR, MMR-deficient status; hMMR, MMR-heterogeneous status; MMR-low, MMR-low status. | | | | | |
